# Implementation of the Infection Risk Scan (IRIS) in nine hospitals in the Belgian-Dutch border region (i-4-1-Health project)

**DOI:** 10.1186/s13756-022-01083-1

**Published:** 2022-02-28

**Authors:** Martine Verelst, Ina Willemsen, Veronica Weterings, Pascal De Waegemaeker, Isabelle Leroux-Roels, Ellen Nieuwkoop, Veroniek Saegeman, Lieke van Alphen, Stefanie van Kleef-van Koeveringe, Marjolein Kluytmans-van den Bergh, Jan Kluytmans, Annette Schuermans, the i-4-1-Health study group, Lieke van Alphen, Nicole van den Braak, Caroline Broucke, Anton Buiting, Liselotte Coorevits, Sara Dequeker, Jeroen Dewulf, Wouter Dhaeze, Bram Diederen, Helen Ewalts, Herman Goossens, Inge Gyssens, Casper den Heijer, Christian Hoebe, Casper Jamin, Patricia Jansingh, Jan Kluytmans, Marjolein Kluytmans-van den Bergh, Stefanie van Kleef-van Koeveringe, Sien De Koster, Christine Lammens, Isabel Leroux-Roels, Hanna Masson, Ellen Nieuwkoop, Anita Van Oosten, Natascha Perales Selva, Merel Postma, Stijn Raven, Veroniek Saegeman, Paul Savelkoul, Annette Schuermans, Nathalie Sleeckx, Krista van der Slikke, Arjan Stegeman, Tijs Tobias, Paulien Tolsma, Jacobien Veenemans, Dewi van der Vegt, Martine Verelst, Carlo Verhulst, Pascal De Waegemaeker, Veronica Weterings, Clementine Wijkmans, Patricia Willemse-Smits, Ina Willemsen

**Affiliations:** 1 Department of Infection Control, University Hospital Leuven, Leuven, Belgium; 2 Department of Infection Control, Amphia Hospital, Breda, The Netherlands; 3 Department of Infection Control, University Hospital Ghent, Ghent, Belgium; 4 Department of Infection Control, Elisabeth TweeSteden Hospital, Tilburg, The Netherlands; 5 Departement of Medical Microbiology, Care and Public Health Research Institute (CAPHRI), Maastricht University Medical Center+, Maastricht, The Netherlands; 6 Laboratory of Clinical Microbiology, University Hospital Antwerp, Antwerp, Belgium; 7 Julius Center for Health Sciences and Primary Care, UMC Utrecht, University Utrecht, Utrecht, The Netherlands

**Keywords:** Infection prevention, Guidelines, Benchmarking, Surveillance

## Abstract

**Background:**

A tool, the Infection Risk Scan has been developed to measure the quality of infection control and antimicrobial use. This tool measures various patient-, ward- and care-related variables in a standardized way. We describe the implementation of this tool in nine hospitals in the Dutch/Belgian border area and the obtained results.

**Methods:**

The IRIS consists of a set of objective and reproducible measurements: patient comorbidities, (appropriate) use of indwelling medical devices, (appropriate) use of antimicrobial therapy, rectal carriage of Extended-spectrum beta-lactamase producing *Enterobacterales* and their clonal relatedness, environmental contamination, hand hygiene performance, personal hygiene of health care workers and presence of infection prevention preconditions. The Infection Risk Scan was implemented by an expert team. In each setting, local infection control practitioners were trained to achieve a standardized implementation of the tool and an unambiguous assessment of data.

**Results:**

The IRIS was implemented in 34 wards in six Dutch and three Belgian hospitals. The tool provided ward specific results and revealed differences between wards and countries. There were significant differences in the prevalence of ESBL-E carriage between countries (Belgium: 15% versus The Netherlands: 9.6%), environmental contamination (median adenosine triphosphate (ATP) level Belgium: 431 versus median ATP level The Netherlands: 793) and calculated hand hygiene actions based on alcohol based handrub consumption (Belgium: 12.5/day versus The Netherlands: 6.3/day) were found.

**Conclusion:**

The Infection risk Scan was successfully implemented in multiple hospitals in a large cross-border project and provided data that made the quality of infection control and antimicrobial use more transparent. The observed differences provide potential targets for improvement of the quality of care.

## Introduction

Healthcare–related infections and antimicrobial resistance pose a global threat to patient-safety. They increase morbidity, length of hospital stay and higher costs of the healthcare system [1]. This also means that efforts regarding infection prevention and judicious use of antimicrobials must be intensified. Prevention of infections is possible by applying bundled basic principles of infection control and implementing an effective antibiotic policy [2]. In real life, making the quality of infection control transparent is difficult and no universal method to measure the quality of care on these aspects is available [2]. Therefore, a standardized method, the Infection Risk Scan (IRIS) was developed by Willemsen et al. [3–5]. The IRIS provides an objective assessment of the quality of infection prevention and antimicrobial use by measuring different patient-and care related risk factors visualized in a risk profile of the patient population and an improvement spider plot.

The objectives of this study are to describe the implementation process of the IRIS in nine cross-border hospitals in Belgium (BE) and The Netherlands (NL) and to present the results of the first IRIS survey, conducted in 2017. The IRIS implementation was part of a larger Interreg project, aiming at broadening the knowledge regarding antimicrobial resistance and use in different healthcare and veterinary settings among cross-border countries, more specifically Belgium and The Netherlands [6]. This paper describes the IRIS-method, its implementation and the differences between both countries.

## Method

### Implementation

The IRIS was conducted in nine hospitals (three Belgian university hospitals, one Dutch university hospital, three Dutch teaching hospitals and two Dutch general hospitals), on 34 hospital wards, from two up to four wards per hospital, depending on the hospital size. In each hospital the IRIS was performed at least on one surgical ward and one internal medicine ward. In addition  a selection was made between one of the following medical specialty’s: urology, cardiology, orthopedics, pulmonology and/or geriatrics. Similar disciplines were selected in order to make comparisons between wards possible.

An expert team, consisting of five infection control practitioners (two with a nursing background and three with a laboratory technician background) with a working experience of at least 2 years in the field of infection control, was established to enable the implementation. A pilot was conducted in two hospitals to fine-tune the instruction manual and the software tool. The software tool allowed the input of data and presented the ward specific risk profile and improvement plot. All local IRIS performers received thorough training by the IRIS experts to improve the standardization of data collection and implementation of the IRIS.

### IRIS risk profile

The risk profile consists of four patient-related variables related to the comorbidity and the susceptibility to healthcare associated infections of the patient population (Table 1). These variables are collected on the day of the IRIS survey:

1. The McCabe score was used as an indication of severity of underlying disease and the remaining life expectancy. This score classifies all patients into four categories: (1) non-fatal or life expectancy more than 5 years, (2) ultimately fatal or life expectancy between 1 and 5 years, (3) rapidly fatal with less than one year life expectance or (4) unknown [7].
2. The presence of indwelling urethral and intravascular (i.e. central and peripheral) devices was included. Suprapubic and epidural catheters were not included. The cut-offs used to classify the risk as high, medium or low were based on prevalence results from (inter)national healthcare associated infections surveillance studies [8].
3. The use of intravenous or oral antimicrobial therapy (AMT) was registered according to the global PPS methodology. Antibiotic beads and cement, antibiotic prophylaxis administrated in the operating theater and topical treatments were not included. The cut-offs for risk classification were based on prevalence results from national surveillance studies [9, 10].
4. The prevalence of rectal carriage of Extended-spectrum beta-lactamase producing *Enterobacterales* (ESBL-E) was measured by culture of faecal, perianal or gastrointestinale stoma swabs. Methods of phenotyping and genotyping are executed as previously reported [11]. The cut-offs for risk classification were based on surveillance report data of the European Centre for Disease prevention and Control (ECDC) [9].

**Table 1:**
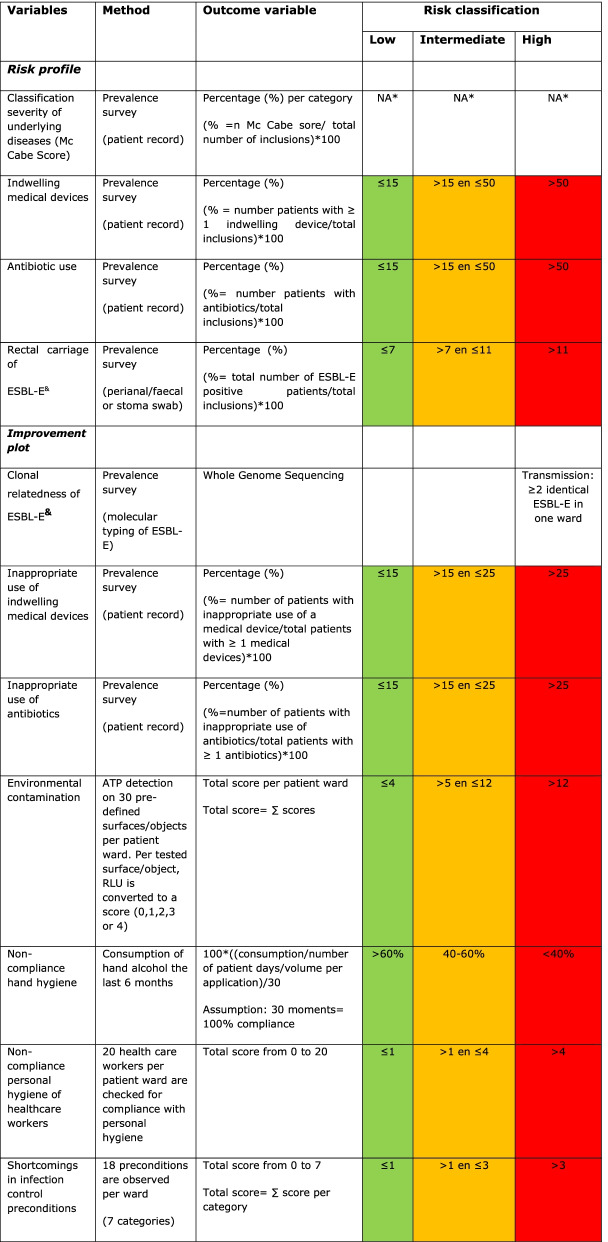
Overview of all collected variables, the method used, outcome variables that are visualized in the risk profile and improvement plot and the breakpoints for the risk classification

*N.A. = Not applicable

^&^ESBL-E = Extended Spectrum beta-Lactamase Enterobacterales

### IRIS improvement plot

The improvement plot represents seven care- and ward- related variables, which are considered important indicators for infection control that can be influenced by the healthcare professionals/organization (Table 1).

1. Clonal relatedness of ESBL-E

Clonal relatedness between ESBL-E was determined based on the similarity of the whole genome multilocus sequence typing (wgMLST) allelic profiles [12, 13]. To determine thresholds for relatedness for this typing scheme, data were gathered from different well-documented bacterial outbreaks, varying in space (different countries over the world) and time (outbreaks lasting from a few days to several years) and involving different sequence types. By combining both sequencing and epidemiological data of these well-described reported outbreaks, similarity thresholds for clonal relatedness were determined (Applied Maths, Belgium, data not published). These thresholds were validated for this study using external quality assessment panels and comparisons between labs [14]. For *Citrobacter* spp., *Enterobacter cloacae*, *Escherichia coli*, *Klebsiella aerogenes*, *Klebsiella oxytoca*, and *Klebsiella pneumoniae*, a similarity percentage of at least 98.00%, 99.43%, 97.54%, 97.70%, 99.13% and 99.64% respectively between the allelic profiles of two strains was used as threshold. For this IRIS survey, when at least two clonally related ESBL-E strains were detected in two or more patients from one ward, this was considered to be indicative for transmission.

2. Appropriate use of indwelling medical devices

**Fig. 1:**
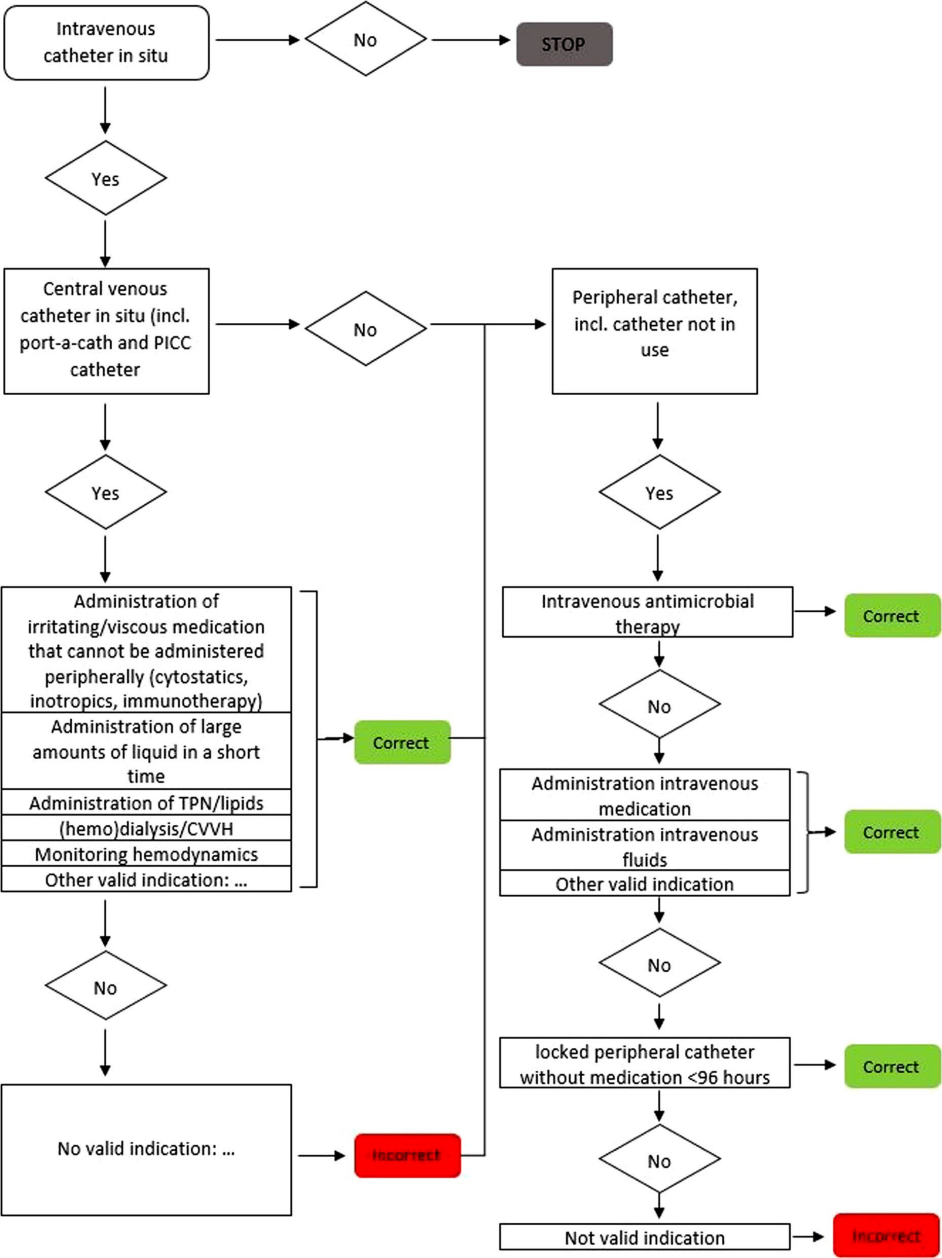

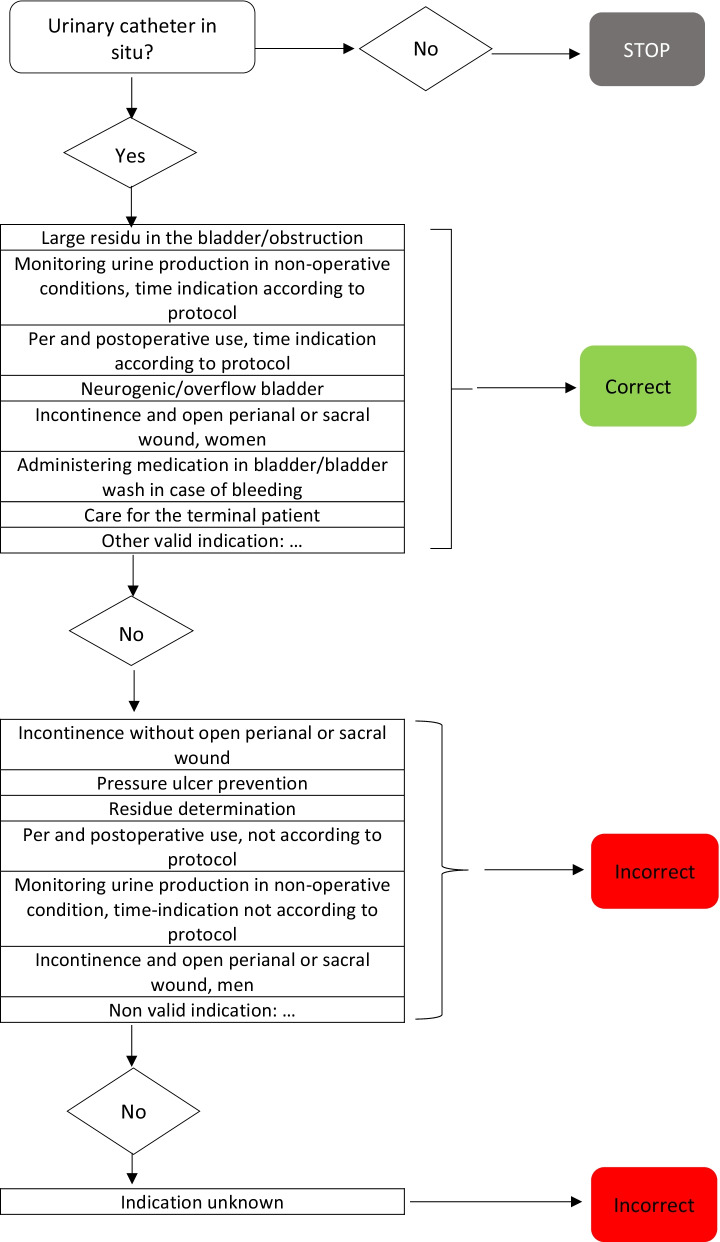
Flowchart appropriate use of indwelling medical devices

The appropriate use of intravascular devices was judged using a standardized flowchart (Fig. 1). The flowchart was developed by the IRIS expert team and is based on national guidelines [15, 16]. The proportion of patients with an inappropriate medical device is presented in the improvement plot. The cut-off points for classification are based on expert opinion as there are no reference values available.

3. Appropriate use of AMT

**Fig. 2:**
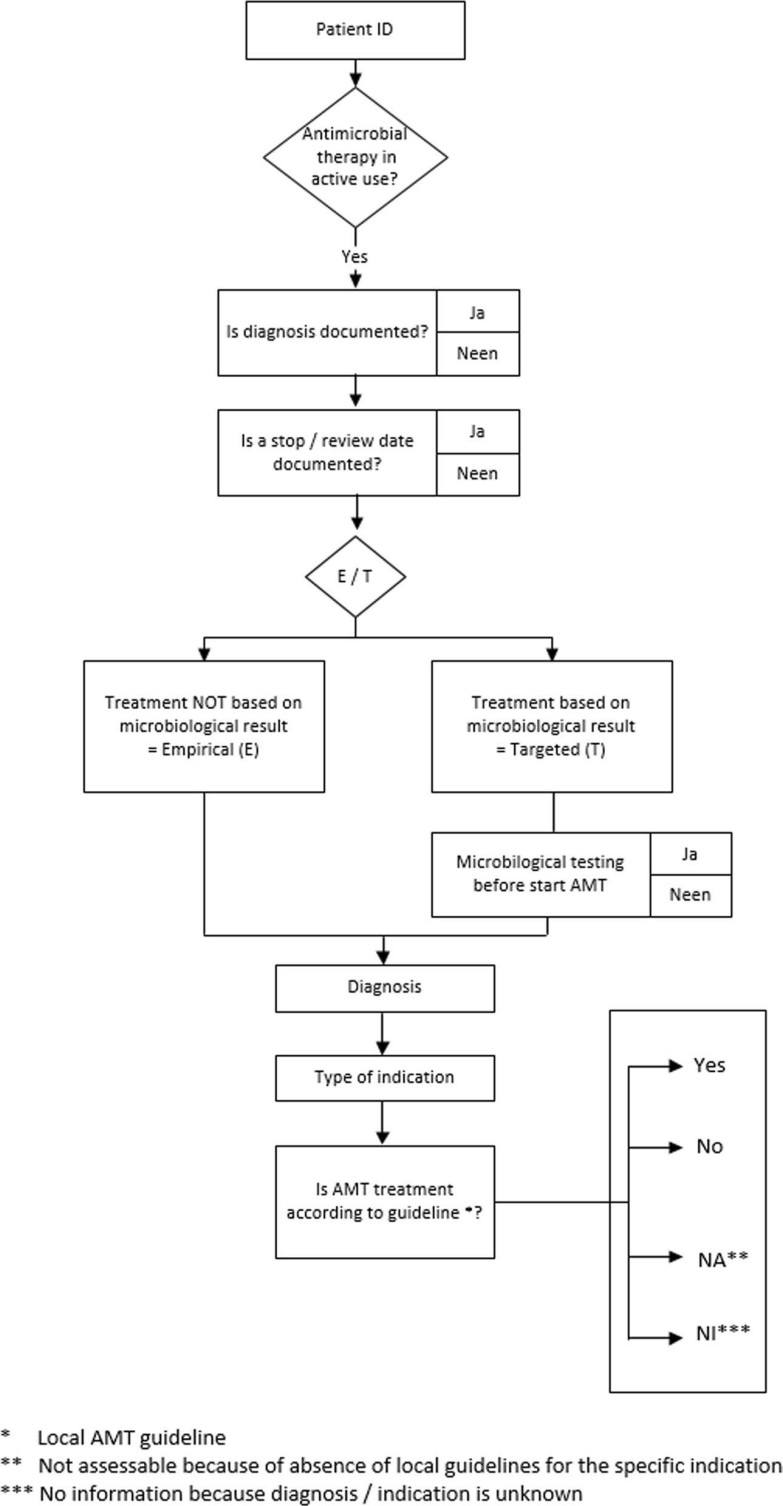
Flowchart appropriate use of antimicrobial therapy

**Fig. 3:**
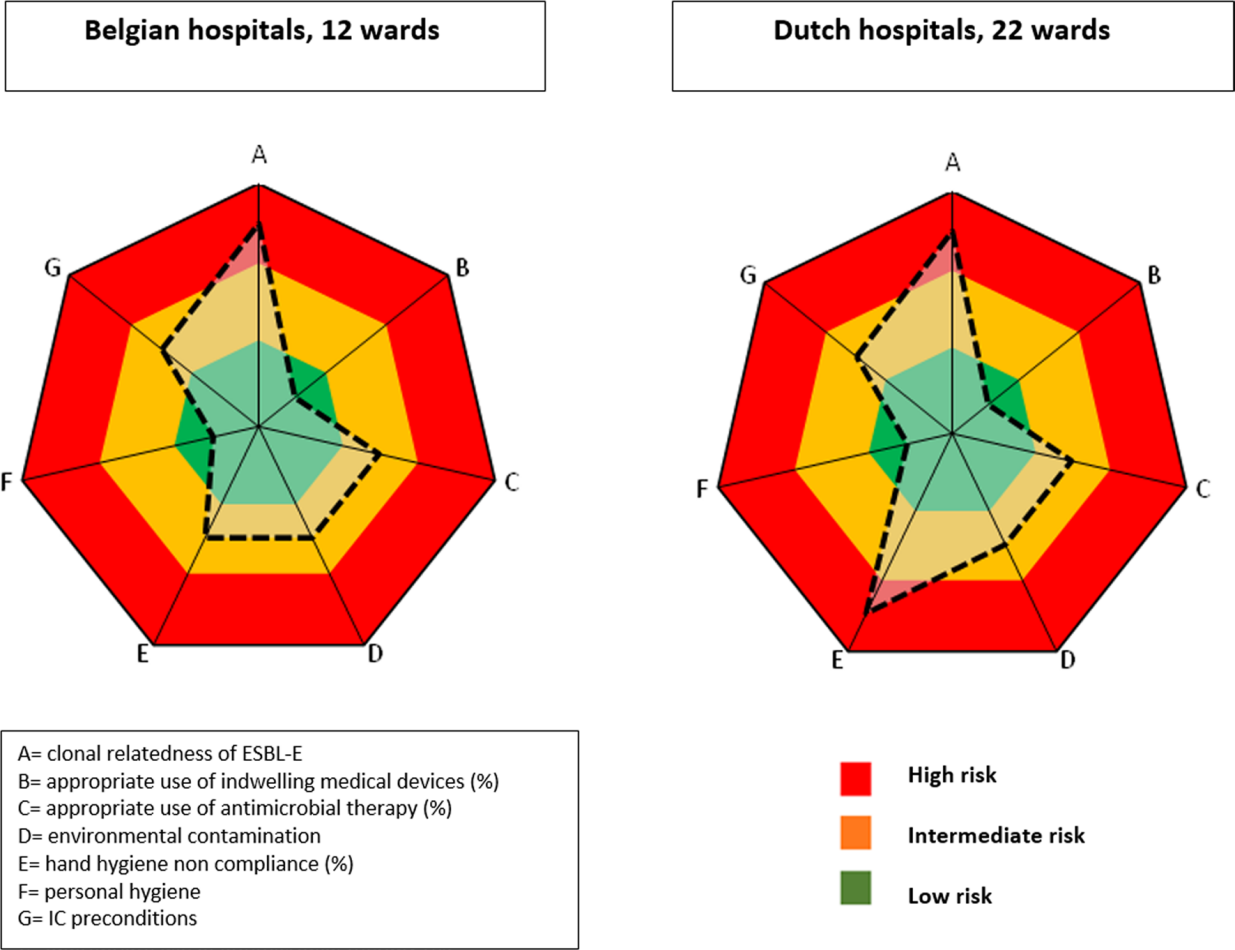
IRIS improvement plots of Belgian and Dutch hospitals

Appropriate use of AMT was judged by a physician or a consultant microbiologist based on the local antibiotic formulary and in accordance with the methodology of Global PPS. Guideline compliance was judged as: according to local guideline, not according to local guideline, no guideline available or insufficient information for judgement (Fig. 2) [17, 18]. The proportion of patients treated with one or more antimicrobials that were considered unjustified according to the local guideline was represented in the improvement plot (Fig. 3).

4. Environmental contamination

To measure the level of environmental contamination the local IRIS performers used an objective technique based on the measurement of adenosine triphosphate (ATP), using the Clean Trace NG luminometer (USA, 3M) [19, 20]. ATP is an indicator for the quantity of organic material present on a surface, expressed in relative light units (RLU). Thirty pre-defined objects and surfaces (10 obligatory and 20 randomly selected) were measured within each patient ward (Table 2). These objects or surfaces were selected based on the following criteria: frequently touched by health care workers; frequently touched by patients; in the direct vicinity of patients; and surfaces for aseptic procedures (e.g. tabletop for medication preparation). The ATP swabs were taken early in the afternoon to standardize the timing. For each test point the result expressed as RLU was converted to a score: < 1000 RLU = 0 points = clean; ≥ 1000 and < 3000 RLU = 1 point = intermediate; ≥ 3000–10,000 RLU = 2 points = contaminated and ≥ 10,000 RLU = 3 points = extremely contaminated [21]. The total score of all tested objects within the unit was presented in the improvement plot as “clean” (green zone), “intermediate” (orange zone) and “contaminated” (red zone). The cut-off points for classification are based on expert opinion.

**Table 2:** Overview of all tested surfaces and objects for environmental contamination on the patient wards

Category	Obligatory/optional	Method 1 or 2*
*Medical devices*		
Blood pressure monitor-control panel	Obligatory	Method 1
Blood pressure monitor-cuff	Optional	Method 1
Thermometer-handle	Optional	Method 2
Glucose meter-control panel	Obligatory	Method 1
Glucose meter-insertion opening	Optional	Method 2
Oxygen saturation meter-measuring probe	Optional	Method 2
Oxygen saturation meter-control panel	Optional	Method 1
Infusion stand	Optional	Method 2
Stethoscope-membrane	Optional	Method 2
Bladder scan-echo head	Optional	Method 2
Bladder scan-control panel	Optional	Method 1
IV pole-control panel	Optional	Method 1
*Patient bound materials*		
Bedrails	Optional	Method 2
Pull-up bracket	Obligatory	Method 2
Patient alarm bell	Optional	Method 2
Overbed table-fixed worktop	Optional	Method 1
Overbed table-extendable worktop	Obligatory	Method 1
Closet-next to handle	Optional	Method 2
*Sanitary items*		
Toilet-seat	Obligatory	Method 1
Toilet-bowl	Obligatory	Method 2
Toilet-support/bracket	Optional	Method 2
Toilet-flush button	Optional	Method 2
Washstand-tap control	Optional	Method 2
Shower-support bracket	Optional	Method 2
Shower-tap control	Optional	Method 2
Shower-showerhead	Optional	Method 2
Toilet seat-sitting area	Obligatory	Method 2
Bedpan cleaner-control panel	Obligatory	Method 1
Washstand-surface around faucet	Optional	Method 2
*Ward bound materials*		
Keyboard-computer on wheels (COW)	Obligatory	Method 1
Keyboard computer-team post	Optional	Method 1
Telephone-handle	Optional	Method 2
Telephone-keyboard	Optional	Method 2
Stool	Optional	Method 1
Work surface cart	Optional	Method 1
Tabletop medication preparation	Obligatory	Method 1
Work surface team post	Optional	Method 1
Chair-seat area	Optional	Method 2
Chair-elbow rest	Optional	Method 2
Railing hallway	Optional	Method 2

*Method 1: surfaces of approximately 100 cm^2^, swabbed in two directions

Method 2: objects without a flat surface or smaller than 100 cm^2^

5. Hand hygiene compliance indicator

Compliance of hand hygiene was based on the consumption of alcohol-based hand rub (ABHR) [21]. The volume of consumed ABHR was divided by the number of patient days covering a period of at least 3 months, divided by the volume of ABHR that is delivered per application (2.5 ml). This results in the number of hand hygiene moments per patient day. In scientific publications the number of hand hygiene opportunities (HHO) per patient and per day in different clinical areas of hospitals range between nine and 78.3 [22–24]. The IRIS expert team and the IRIS performers defined a compliance rate of 100% when 30 HHO were performed per day, per patient.

6. Personal hygiene of health care workers (HCW)

At least 20 HCW (e.g. nurses, physicians, cleaning staff, physiotherapists, …) per ward were each observed by the IRIS performer to evaluate their compliance with basic hygiene rules: absence of watch or wrist jewelry, no rings, forearms uncovered, no artificial nails, no nail polish, tied hair, short beard, headscarf tied together [25, 26]. The total score of all HCW is presented in the spider plot (Fig. 3).

7. Shortcomings in infection prevention preconditions

Several prerequisites are essential for an effective infection control policy. The selection of the preconditions, listed in Table 3, was made by the IRIS expert team based on Joint Commission Standards [27] and on expert opinion. Scores were given in case the preconditions per category items were present or absent.

**Table 3:** Overview of the infection control preconditions

Infection control preconditions
**1. Personal protective equipment is present** Gloves are present at every point of care* Aprons are present at the patient ward Surgical masks are present at the patient ward
**2. A bedpan washer-disinfector is available and meets following requirements** Disinfection with steam or hot water of at least 80° for at least 60 s Bedpan washer or shredding system has been validated
**3. Hand hygiene is possible at every point of care** There is hand alcohol available at every point of care There is hand soap available at every point of care There are only disposable towels or wipes available at the patient ward There is a hand hygiene poster present at the patient ward
**4. Document management system of infection prevention protocols is available** 3 nurses from the ward are able to find 2 protocols regarding an infection prevention subject e.g. hand hygiene, isolation precautions
**5. Clean-dirty separation** Linen is stored dust-free and kept away from moisture Clean and dirty linen are processed separately There is a visual separation of a clean and dirty zone in the utility
**6. Existing chairs or benches can be easily cleaned and disinfected** There are no fabric chairs or couches in the ward The upholstery of chairs and benches is intact
**7. An expiration date/period of use of patient-bound materials is not exceeded** Check the expiring date of at least 5 objects (ex. syringe, blood tubes) Check the expiring date of at least 5 products (care products, skin antiseptics)

^*^Point of care = place where 3 elements are present: the patient, the health care worker and the care or treatment of the patient

For each variable in the risk profile and in the improvement plot, breakpoints were set to make the division in three categories: low, intermediate and high risk or improvement potential. Breakpoints were based on national prevalence surveys, scientific publications or expert opinion. Table 1 provides an overview of the included variables, methods, score system, outcome measures and risk classifications.

### Data collection

Several point prevalence IRIS surveys were performed by the trained IRIS performers, from all the participating hospitals using the standardized electronic record forms. To achieve sufficient datapoints at least 50 patients per ward had to be included in each IRIS.

### Statistical methods

Data were analyzed with Statistical Package for Social Science software (SPSS; IBM Corp, Armonk, New York, US; version 27).Categorical variables were analyzed by the Pearson χ^2^ test and ordinal variables were analyzed using the Mann–Witney U test. Statistical significance was accepted when p-value was < 0.05.

## Results

### IRIS in Belgian hospitals

#### Risk profile (Table 4)

**Table 4:** Patient characteristics in Belgian and Dutch hospitals

	Belgian hospitals	Dutch hospitals	*P* value
Female	48.1% (298/619)	46.7% (514/1098)	> 0.05
Median age (years)	66	71	< 0.001
Range	18–97	19–103	
IQR	IQR = 25.5	IQR = 19	
Ward specialty			< 0.05
Surgery	47.2% (292/619)	54.4% (598/1098)	
Medicine	52.8% (327/619)	45.5% (500/1098)	
McCabe score			> 0.05
Non fatal (> 5 years)	78.5%(486/619)	79.1% (869/1098)	
Eventually fatal (1–5 years)	19.4%(120/619)	13.5% (148/1098)	
Fatal within 1 year	1.9% (12/619)	2.3% (25/1098)	
Unknown	0.02% (1/619)	5.1% (56/1098)	
Indwelling medical devices	63.3% (392/619)	66.2% (727/1098)	= 0.186
Urinary catheter	18.1% (112/619)	19.9% (219/1098)	
Intravenous catheter	58.2% (360/619)	60.7% (666/1098)	
Missing	0.03% (2/619)	0.08% (9/1098)	
Antimicrobial therapy	41.5% (257/619)	42.7% (469/1098)	> 0.05
Rectal carriage ESBL-E	15.0% (92/619)	9.6% (105/1098)	< 0.05

*NS* not significant, *SD* standard deviation, *IQR* interquartile range

A total of 619 (58%) patients of the 1060 patients hospitalized in 12 different wards were included from three hospitals. Medical specialties were surgery (n = 292; 47.2%) and internal medicine (n = 327; 52.8%). The median age was 66 years (IQR = 25.5). The Mc Cabe scores for 619 patients resulted in: 486 patients (78.5%) non-fatal, 120 patients (19.4%) eventually fatal and 12 patients (1.9%) fatal within one year. Of all patients, 392 (63.3%) had an indwelling medical device. A total of 257 (41.5%) patients received AMT and of those, 187 (72%) were treated with one antibiotic, 53 (20%) with two antibiotics and 21 (8%) received three or more antibiotics. Of all patients, 92 (15%) were rectal carrier of ESBL-E and of those 19 swabs (21%) were taken less than 48 h after hospital admission. ESBL was mainly detected in *Escherichia coli* (n = 61; 66%), *Enterobacter cloacae* (n = 10; 11%) and *Klebsiella pneumoniae* (n = 9; 10%).

#### Improvement plot (Fig. 3)

With regard to clonale relatedness, a total of 10 clusters of ESBL-E were detected on different wards; respectively one cluster of *Klebsiella pneumoniae *(2 patients), one cluster of *Citrobacter *spp. (2 patients), one cluster of *Klebsiella oxytoca *(2 patients), one cluster of *Enterobacter cloacae* complex (2 patients) and 6 clusters of *Escherichia coli *(range of 2–3 patients).

Of all patients with one or more devices in situ, 38 (9.7%) were assessed as inappropriate. Of all urinary catheters, 18 (16.1%) were considered unjustified according to the standardized flowchart. Forty-eight patients (19%) were receiving AMT that was not consistent with the local guidelines.

Regarding ATP measurements, to assess environmental contamination, a total of 726 items were tested; 596 items (82%) were considered clean, 94 intermediate (13%), 22 contaminated (3%) and 14 extremely contaminated (2%). The median RLU-value measured was 431 [21].

In each ward a hand alcohol product was used. An amount of 2052 l of hand disinfectant was consumed in the 12 investigated wards with in total 65,585 patient days resulting in 31.3 ml of hand disinfectant. Considering a volume of 2.5 ml per application, a total of 12.5 hand hygiene actions per patient day were calculated.

Two hundred forty HCW (120 nurses, 36 physicians, 32 paramedics and 52 others) were tested for the basic personal hygiene rules. Ten HCW (4.2%) did not meet the basic requirements.

With regard to infection control preconditions, in five wards linen was not stored dust-free, in four wards fabric chairs were present and in seven wards products were expired. Additionally, a hand hygiene poster was absent in one ward and there was no separation between clean and dirty zone in the utility.

### IRIS in Dutch hospitals

#### Risk profile (Table 4)

A total of 1098 patients (67%) of the 1630 patients hospitalized in 18 different wards were included in six hospitals. Medical specialties were surgery (n = 598; 54.5%) and internal medicine (n = 500; 45.5%). The median age was 71 years (IQR = 19). The Mc Cabe scores for 1098 patients were: 869 patients (79.1%) non-fatal, 148 patients (13.5%) eventually fatal and 25 patients (2.3%) fatal within one year. Of all patients, 727 (66.2%) patients had an indwelling medical device. A total of 469 (42.7%) patients were on AMT and of those, 342 (73%) were treated with one antibiotic, 111 (24%) with two antibiotics and 16 (3%) received three or more antibiotics. Of all patients, 105 (9.6%) were carriers of ESBL-E. Of those, 10 swabs (9.5%) were taken less than 48 h after hospital admission. ESBL was mainly detected in *Escherichia coli* (n = 59; 56%), *Klebsiella pneumoniae* (n = 35; 33%) and *Enterobacter cloacae* (n = 7; 7%).

#### Improvement plot (Fig. 3)

With regard to clonale relatedness, a total of seven clusters of ESBL-E were detected on different wards; respectively two clusters of *Klebsiella pneumoniae *(2 patients) and five clusters of *Escherichia coli* (2 patients).

Of all patients with one or more invasive device in situ, 37 (5.1%) were assessed as inappropriate. Of all urinary catheters, 24 (11.0%) were considered unjustified according to the local protocol. Eighty-seven patients (18.9%) were receiving AMT that was not consistent with the local guidelines.

Regarding environmental contamination, a total of 1288 items were tested, 876 (68.0%) were considered clean, 268 (21.0%) intermediate, 98 (8.0%) contaminated and 46 (3.5%) extremely contaminated. The median RLU-value measured was 793 [21].

In each ward, a hand alcohol product was used, dispensing 2.5 ml per application. The volume of ABHR consumed during a period of at least 3 months was 1094 l in 18 investigated wards. Giving the 69,143 patient days in these period on these wards, this resulted in 15.8 ml of hand disinfectant per patient day. Assuming a volume of 2.5 ml per application, a total of 6.3 hand hygiene actions per patient day was calculated. One hospital was unable to provide data regarding the consumption of hand alcohol.

Four hundred fifty-three HCW (254 nurses, 80 physicians, 27 paramedics and 92 other) were tested for the basic hand hygiene rules. Nineteen HCW (4.2%) did not meet the basic requirements.

Considering the infection control preconditions, in six wards linen was not stored dust-free, in 13 wards fabric chairs were present, in one ward the products were expired. Additionally, a hand hygiene poster was absent in seven wards and there was no separation between clean and dirty zone in the utility in six wards.

### IRIS as a benchmark tool between countries

#### Risk profile

Significant differences were found in age, ward specialty and ESBL-E carriage. No significant differences were detected in the prevalence of indwelling medical devices or McCabe score (Table 4).

#### Improvement plot (Fig. 3)

Clonal relatedness of ESBL-E: A total of 17 clusters was found, seven clusters of two patients in Dutch hospitals; nine clusters of two patients and one cluster of three patients in Belgian hospitals. Clusters were spread over five different Belgian and six different Dutch wards. In eleven ESBL-E clusters *Escherichia coli* was involved*.*

Indwelling medical devices: Overall, there was no significant difference in the presence of indwelling medical devices. However the type of medical device and the indications for usage differed between BE and NL.

Antimicrobial use: No significant difference in the use of AMT or inappropriate use of antimicrobials between countries was found.

Environmental contamination: A total of 2,014 ATP measurements were performed in the participating hospitals. The median RLU measured in Dutch hospitals was 793 and significantly differed (*p* < 0.001) from the median RLU (431) in Belgium [21].

Furthermore, the number of HHO per patient day was 12.5 in Belgian hospitals and 6.3 in Dutch hospitals. The hand hygiene indicator revealed higher compliance in Belgian hospitals (*p* < 0.05).

Overall, an equal percentage (96.8%) of the observed HCW met the conditions of personal hygiene in BE and NL.

Overall, no significant differences were found in infection prevention preconditions between the two countries.

### IRIS as a benchmark tool between wards

**Fig. 4:**
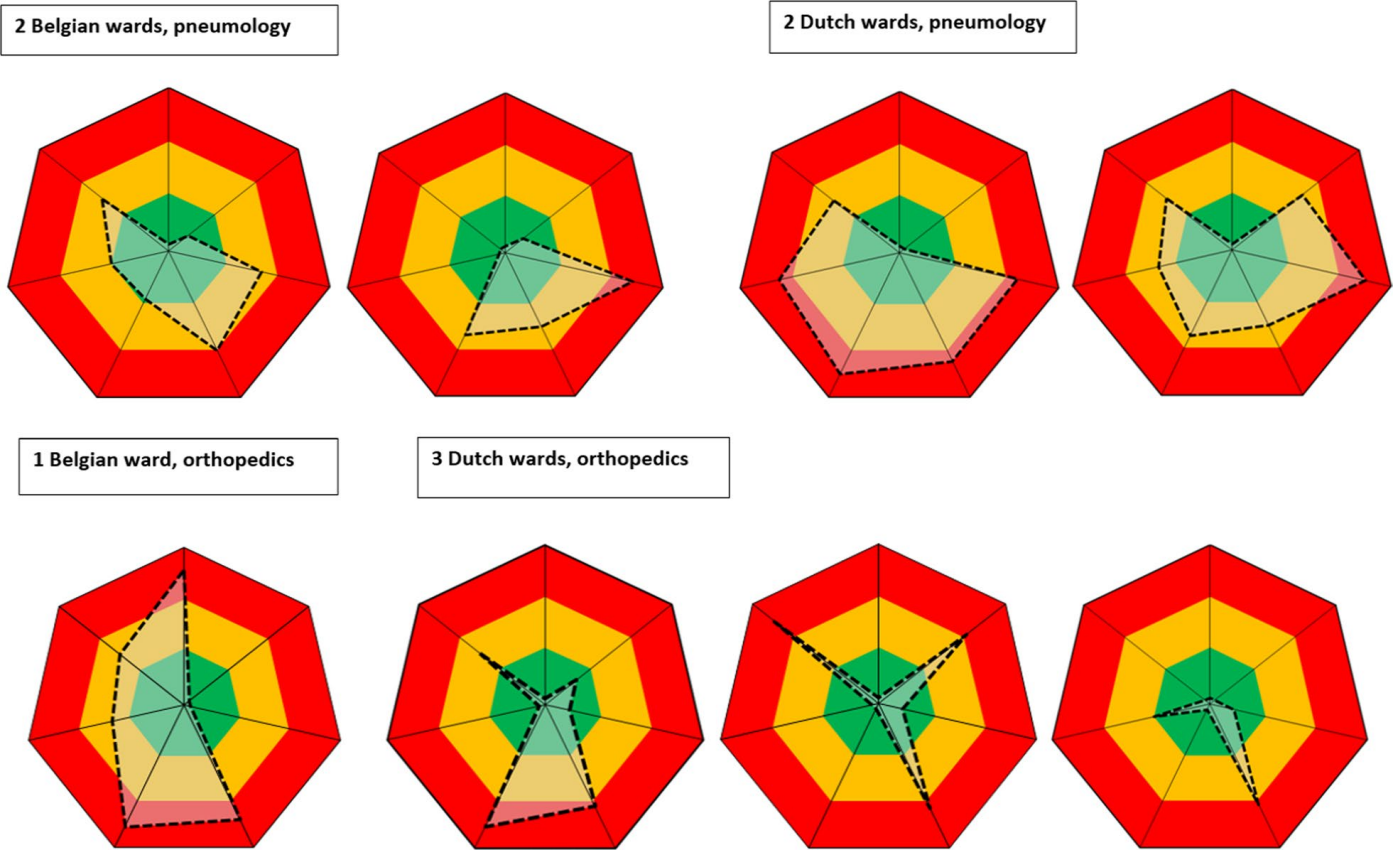
Improvement plot of different wards in Belgian and Dutch hospitals

The IRIS visualizes local performances on infection control and AMT and enables to compare different patient wards. Figure 4 represents several improvement plots of different disciplines and reveals different improvement opportunities on various ward-and care related variables. Some care-and ward related variables are similar between the identical medical specialties. For two belgian pneumology wards the appropriateness of indwelling medical devices was similar and implied a low risk on the improvement plot. However, some ward related variables display a substantive variation revealing that each ward requires specific improvement programs.

## Discussion

We demonstrated the feasibility of implementing the IRIS tool in nine Belgian and Dutch hospitals. This implementation was supported by an active training program of the local HCW by a team of experts. The active training program included education about the patient data collection, performance of ATP measurements and gathering data about all variables of the improvement plot. By using this method, the following advantages became apparent. First, the bundle approach provides a complete picture of the quality of infection prevention and antimicrobial use. Second, implementing the IRIS promotes interdisciplinary cooperation. Third, results are visualized using color codes in an easy to understand spider plot. Fourth, the results provide targets for interventions and the effects can be measured by repeated measurements. Finally, the multifactorial measurements are relatively objective and reproducible which it a benchmark tool between healthcare settings. In this study following results became apparent:

Significant differences in the prevalence of ESBL-E carriage were detected between the two countries (BE: 15.0% versus NL: 9.6%). Additionally, the prevalence observed in samples taken less than 48 h after hospital admission differed between BE (n = 19; 21%) versus NL (n = 10; 9.5%) suggesting a higher ESBL-E carriage in the Belgian community. Recent studies reported higher ESBL carriage rates in BE (11.6%) [28] than in the Dutch community (8.6%) [29, 30]. The differences in infection control policies and antimicrobial use may explain the difference in ESBL-E carriage between the two countries. Remark that the results of molecular typing need to be interpreted with caution. As typing data in the IRIS could not be combined with extensive epidemiological data for interpretation, strict thresholds for clonal relatedness were applied which could result in an underestimation of transmission. In addition, the finding of clonally related strains does not necessarily imply transmission within the healthcare institution, but may also reflect the influx of a successful clone or clones from the community [30].

There was no difference in the use of AMT in the Belgian versus the Dutch hospitals. The relatively high AMT consumption can be explained by the selection of the wards (lung diseases, abdominal surgery, geriatrics) where infectious pathologies are highly prevalent. The proportion of inappropriate AMT (BE: 19.0% versus NL: 18.9%) was equal in both countries but higher than the target set by BAPCOC (10% inappropriate AMT according to local guidelines) [28]. This stresses the need for improvement in antibiotic stewardship- programs. Because local guidelines may differ between institutions, this variable is less suitable for comparising hospitals and countries.

The level of environmental contamination was higher in the Dutch hospitals. Cleaning staff (internal versus external) and different cleaning protocols possibly explain these results. In Dutch hospitals, medical devices, patient bound materials and sanitary items are cleaned using microfiber cloths. Additional disinfection is executed only in case of contamination with blood or body fluids or after discharge of patients carrying multidrug resistant microorganisms. In Belgian hospitals, patient bound materials and sanitary items are daily cleaned. High-risk surfaces are disinfected using disinfectant wipes. Further studies need to confirm if this can explain the observed differences.

Hand hygiene was measured using consumption volumes of hand alcohol. These data are easy to generate and are reproducible at hospital and ward level. Moreover, there is no observer bias and it is less time-consuming than performing direct observations. Nevertheless, there are a few disadvantages of our method. Consumption of ABHR does not always represent use by HCW but also patients and visitors can use hand alcohol. Dispensers are sometimes given to the patient when leaving the hospital or left-overs are thrown away. To obtain 100% compliance we agreed upon a limit of at least 30 hand hygiene moments that should be performed per day, per patient. Although this assumption was set for all wards, we realize that in fact the number of hand hygiene moments differs between specialties. The hand hygiene indicator revealed significantly higher results in the Belgian hospitals. We attribute this difference to more awareness on hand hygiene in Belgium with e.g. the national campaigns for hand hygiene aiming to create a sustainable change in hand hygiene compliance. Education, audit and feedback take a significant part in these campaigns. The results consistently confirm improved compliance rates of hand hygiene after running the campaign [31].

Personal hygiene of HCW revealed no significant differences between the two countries. This result is not surprising, since there is no difference in dress code and jewelry policy between the two countries.

The infection prevention preconditions are comparable between the two countries. This is in accordance with guidelines for standard precautions to prevent the transmission of microorganisms. Both Dutch and Belgian guidelines on standard precautions are based on the Centers for Disease Control and Prevention, World Health Organization guidelines and the Joint Commission International standards.

The IRIS also entails a few limitations: executing the IRIS is time consuming and requires efforts from different healthcare workers: nurses for informing and sampling patients; infection control practitioners for collecting patient records, taking ATP swabs, assessing the use of indwelling medical devices, summarizing infection control preconditions and clothing regulations; laboratory technicians for analyzing the swabs; microbiologists and physicians for assessing the appropriateness of AMT. The time needed for the IRIS performers to collect data was largely dependent on how the (electronic) patient records were organized. Digitalization of processes can facilitate data collection and ideally it could be largely automated. Also the thresholds can be debated. They were based on data from surveillance programs or peer-reviewed publications. When evidence was lacking or no references were available, thresholds were chosen arbitrarily by the IRIS expert team. The threshold values should be evaluated periodically and adjusted when necessary. Although this might complicate the comparison of results between hospitals.

## Conclusion

In conclusion, the IRIS was implemented successfully in nine hospitals and revealed important differences but also similarities in infection control and antimicrobial use between hospitals. These results can be used to improve the quality of care and repeated measurements can be used to monitor the effects of interventions both on the ward and institutional level. Also, they create transparency to patients and other stakeholders about the safety in hospitals.

## Data Availability

All data were anonymized, i.e. data cannot be directly or indirectly related to their source. Data on institutions were and farms are pseudonymised, I.E. identifying information is replaced by a code and a key file that links this code to the identifying information is kept separate from the research data.

## Abbreviations

ABHR: Alcohol based hand rub
AMT: Antimicrobial therapy
ATP: Adenosine triphosphate
BE: Belgium
ECDC: European Centre for Disease prevention and Control
ESBL-E: Extended spectrum beta-lactamase producing Enterobacterales
HCW: Health care workers
HHO: Hand hygiene opportunities
IRIS: Infection Risk Scan
IQR: Interquartile range
NL: The Netherlands
RLU: Relative light units
SD: Standard deviation
wgMLST: Whole genome multilocus sequence typing
